# Increase of radiologically determined muscle area in patients with liver cirrhosis after transjugular intrahepatic portosystemic shunt

**DOI:** 10.1038/s41598-023-43938-6

**Published:** 2023-10-10

**Authors:** Christine March, Maximilian Thormann, Sarah Geipel, Jan-Peter Sowa, Felix Barajas Ordonez, Maciej Pech, Jazan Omari, Peter Lemmer

**Affiliations:** 1 Department of Radiology and Nuclear Medicine, Otto-Von-Guericke University Magdeburg, Leipziger Str. 44, 39112 Magdeburg, Germany; 2 Department of Medicine, Universitätsklinikum Knappschaftskrankenhaus Bochum, Ruhr University, Bochum, In der Schornau 23-25, 44892 Bochum, Germany; 3 Department of Diagnostic and Interventional Radiology, University Hospital RWTH Aachen, Pauwelsstraße 30, 52074 Aachen, Germany; 4 Department of Gastroenterology, Hepatology, and Infectious Diseases, Otto-Von-Guericke University Magdeburg, Leipziger Str. 44, 39112 Magdeburg, Germany; 5 Department of Radiology and Nuclear Medicine, University of Magdeburg, Leipziger Str. 44, 39120 Magdeburg, Germany

**Keywords:** Gastroenterology, Hepatology

## Abstract

Sarcopenia is common in patients with liver cirrhosis and related to higher mortality. Implantation of a transjugular intrahepatic portosystemic shunt (TIPS) is a feasible method for reducing cirrhosis-related portal hypertension, but also possible improvement of the patient`s muscle status. We aimed to analyze changes in muscle quantity and prevalence of sarcopenia after TIPS. We retrospectively surveyed the muscle status in 52 patients (mean age 54.2 years) before and after TIPS by evaluating skeletal (SMI) and psoas muscle indices (PMI) in CT and MR images. Model for End-Stage Liver Disease (MELD), Freiburg index of post-TIPS survival (FIPS), and their underlying laboratory parameters (e.g., Albumin) were analyzed. Prevalence of sarcopenia was 84.6%. After a median follow-up of 16.5 months after TIPS, SMI (0.020) and PMI (*p* < 0.001) increased, and sarcopenia decreased by 14.8% (0.109). MELD and PMI after TIPS were negatively correlated (r = − 0.536, *p* < 0.001). Albumin levels increased in patients with increased SMI after TIPS (*p* = 0.022). Confirming the positive impact of TIPS implantation on muscle indices in patients with liver cirrhosis, we found indications for improved survival and possible indications for altered metabolism with increased albumin levels in patients with increased muscle quantity.

## Introduction

Regardless of the etiology, liver cirrhosis is the common end stage of chronic liver diseases^1^, including alcoholic liver disease (ALD), non-alcoholic liver disease (NAFLD), chronic hepatitis B and C, autoimmune liver disease, hemochromatosis, and Wilson’s disease. The prevalence of etiologies differs between world regions. In Asia and Africa, liver cirrhosis develops predominately after infection with hepatitis B or C. In contrast, the leading causes in Europe and America are NAFLD and ALD^2,3^.

The prognosis of liver cirrhosis depends on the severity of hepatic dysfunction^4^. In daily practice, the Child–Pugh and the model for end-stage liver disease (MELD) score are generally applied as the main prognostic tools^5^. However, both scoring systems lack important parameters regarding the nutritional status of patients, as liver cirrhosis is strongly related to the development of malnutrition and sarcopenia^6^.

Sarcopenia and malnutrition are known to have an impact not only on the morbidity and quality of life^6,7^ but on survival in patients with liver cirrhosis^8^. According to the European Working Group on Sarcopenia in Older People (EWGSOP2), sarcopenia is defined as a generalized and progressive skeletal muscle disorder^9^ and is associated with malnutrition^10^. The diagnosis is made by evaluating low muscle strength and low muscle quantity or quality. The development of malnutrition and sarcopenia in patients with liver cirrhosis is caused by altered protein metabolism, which leads to increased muscle depletion. Notably, the prevalence of sarcopenia is different among different entities of liver cirrhosis. Patients with alcoholic liver disease tend to have a higher prevalence of sarcopenia than patients with other causes of liver cirrhosis^11^.

According to the American Association for the Study of Liver Diseases, the gold standard for assessing sarcopenia in patients with liver cirrhosis is CT imaging with the skeletal muscle index derived from measuring the height-normalized skeletal muscle area at L3. Hence, low skeletal muscle mass and sarcopenia are usually used synonymously, although muscle strength is rarely measured in studies on sarcopenia in patients with liver cirrhosis^6^.

Tissue inflammation within the liver parenchyma leads to the remodeling of regular liver parenchyma into fibrotic tissue and, subsequently, the development of portal hypertension^2^. In decompensated cirrhosis, this drives the formation of therapy-refractory ascites and variceal hemorrhage due to excessive varicosis. In these cases, a transjugular intrahepatic portosystemic shunt (TIPS) is a feasible treatment option^12^ to lower portal hypertension and, thus, ascites and the risk for variceal hemorrhage. The latest data suggest that the implantation of a TIPS not only lowers portal hypertension but may also improve body composition, specifically muscle status, and thus reduce morbidity and mortality^10,13–19^.

We conducted this study to evaluate changes in skeletal muscle mass (SMM) after TIPS implantation using the PMI and SMI and compared subgroups of cirrhotic patients regarding their etiology of cirrhosis. We also compared the psoas muscle index (PMI) and skeletal muscle index (SMI) and analyzed the correlation with the MELD (Model for End-Stage Liver Disease) and FIPS (The Freiburg index of post-TIPS survival).

## Materials and methods

### Patient selection

In this retrospective study, we included patients with liver cirrhosis who underwent a TIPS procedure at the Department of Radiology and Nuclear Medicine, Otto-von-Guericke University Magdeburg, between April 2010 and April 2021. The majority of cases (94.2%) comprised elective procedures due to therapy-refractory ascites. A few patients underwent an emergency procedure due to severe, otherwise not manageable bleeding. All patients gave verbal and written consent for the procedure. In addition, it was essential for the patients to undergo a minimum of one computed tomography (CT scan) or magnetic resonance imaging (MRI), both prior to and following the TIPS procedure, to assess the PMI and SMI. We only included patients with imaging at least six months after TIPS.

Exemplary indications for the performance of a CT or MRI were planning of the TIPS procedure, search for hepatocellular carcinoma, evaluation of liver cirrhosis, portal vein thrombosis, and evaluation for liver transplantation.

The institutional review board approved the retrospective data analysis and waived specific further consent from patients (Number: 145/21, Ethics Committee, University of Magdeburg, Magdeburg, Germany). All methods were performed in accordance with the relevant guidelines and regulations.

### TIPS procedure

The TIPS procedure was performed under local anesthesia with an ultrasound-guided puncture of the right internal jugular vein, followed by the insertion of a 10F catheter sheath. This was followed by the catheterization of the right hepatic vein. Simultaneously, an assistant conducted an ultrasound of the liver with the imaging of the right hepatic and central portal veins. After confirmation of the correct position, the portal vein was directly punctured through the liver parenchyma. Next, a guidewire and pigtail catheter were inserted to stabilize and confirm the intraportovenous position. After administration of 7.5 mg Piritramid i.v., a balloon catheter was inserted to dilate the intervascular parenchyma. Finally, a Luminexx® stent with 8 or 10 mm diameter or GORE® Viatorr® TIPS endoprosthesis with 10 mm diameter was implanted. The stents were 40–100 mm long. After stent or stent-graft implantation, the portal pressure gradient was measured via a pigtail catheter in the central portal vein and the right atrium. A Shaldon catheter was inserted in the right internal jugular vein for easy access and possible further dilation of the stent during the following days.

### Image acquisition and analysis

For the evaluation of muscle area and indices, we analyzed (contrast-enhanced) computed tomography (CT, 128-section scanner, Somatom, Siemens® or 80-section scanner, Toshiba®) or magnetic resonance imaging (MRI, 1.5 or 3.0 T, Philips®) from one to three months before the TIPS procedure. CT or MRI images were usually obtained for the evaluation of complications arising from cirrhosis or for the exclusion of hepatocellular carcinoma. After TIPS, CT and MR images from at least six months post-procedure were analyzed. First, the areas of the right and left psoas major muscle at the L3 inferior endplate level in the CT and MR images were delineated by hand using Infinitt® PACS Viewer. In the second step, we used AsanJ-Morphometry software (Asan Image Metrics, Seoul, Korea) for semi-automated adipose and muscle tissue segmentation at the cross-sectional area at the L3 inferior endplate level. The segmentation comprises an estimation of total fat area (TFA), skeletal fat area (SFA), visceral fat area (VFA), total muscle area (TMA), total muscle fat area (TMFA), and area of right and left psoas major muscle (PMA). For further analysis, we focused on the psoas muscle area (PMA) and total muscle area (TMA), which comprises the complete muscle area of the cross-sectional L3 endplate level. For semi-automated segmentation, we used predefined thresholds for Hounsfield units (HU) on CT (> − 30 HU and ≤ 150 HU) and the signal intensity (SI) on pre-contrast T1-weighted MRI (> 350 SI and ≤ 750 SI) for muscle and adipose area (≤ − 30 HU in CT and > 100 SI and ≤ 350 SI in MR images)^20^.

Two readers with five years of experience in abdominal imaging, respectively, conducted the delineation and segmentation.

### Analysis of clinical parameters

For the calculation of the MELD^21^ and FIPS^22^ score, we gathered laboratory parameters (creatinine, bilirubin, INR, albumin) from the period of the pre-interventional and post-interventional imaging. The MELD score includes creatinine, bilirubin, and INR to evaluate the severity of liver disease. It was initially developed to predict the survival of patients undergoing TIPS^21^. The FIPS was developed to identify patients with a high risk of post-interventional mortality and includes creatinine, bilirubin, albumin, and the patient’s age. A FIPS score of ≥ 0.92 (85th percentile) is associated with a significantly decreased survival rate after TIPS implantation^22^. A FIPS of ≥ 0.64 was also confirmed as a relevant prognostic cut-off value^23^. We also separately analyzed changes in creatinine, albumin, and bilirubin levels.

### Statistical analysis

We used IBM SPSS Statistics 26.0® (IBM Corp., Armonk, NY, USA) and Excel (Microsoft®, Office16) for statistical analysis. Patients’ characteristics were evaluated using descriptive statistics. We used the Mann–Whitney U test to evaluate muscle area and indices between groups and performed Pearson’s correlation analysis with PMI, SMI, MELD, and FIPS. Categorical variables were tested with the Chi-squared test. For the evaluation of muscle area changes over time, we used the McNemars test.

All tests were carried out two-sided, and a *p*-value ≤ 0.05 was considered statistically significant.

## Results

### Patient characteristics

In this retrospective study, we included 52 patients (42 males, 10 females) with a mean age of 54.2 years (± 10.3) who had undergone TIPS procedures for complications of liver cirrhosis between 2010 and 2021. Furthermore, all of them had received a CT or MR imaging before and after the intervention (Table 1).

**Table 1:** Characteristics of 52 patients who had undergone TIPS procedure.

	n = 52
Sex	
Male, %	42 (80.8)
Female, %	10 (19.2)
Age (years), mean ± SD (range)	54.2 ± 10.3 (14–73)
Time of follow-up (months), median (range)	16.5 (6–93)
Origin of liver cirrhosis	
Alcohol, %	36 (69.2)
NASH, %	4 (7.7)
Autoimmune, %	2 (3.8)
Other, %	5 (9.6)
Child–Pugh-Score	
A, %	3 (7.7)
B, %	26 (66.7)
C, %	10 (25.6)
Ascites, %	42(80.8)
Gastropathy, %	42 (80.8)
Indication TIPS	
Therapy-refractory ascites, %	39 (75.0)
Variceal hemorrhage, %	11 (21.2)
Other, %	2 (3.8)
Urgency	
Emergency, %	3 (5.8)
Elective, %	49 (94.2)
FIPS-Index	− 0.3 ± 1.7 (− 5.74 to 1.29)
Overall survival 1 month prediction (%), mean ± SD (range)	95.0 ± 3.6 (84.5–100.0)
Overall survival 3 months prediction (%), mean ± SD (range)	85.6 ± 9.7 (59.1–100.0)
Overall survival 6 months prediction (%), mean ± SD (range)	80.1 ± 12.8 (46.4–99.9)

NASH—nonalcoholic steatohepatitis; HCC—hepatocellular carcinoma; TIPS—transjugular intrahepatic portosystemic shunt; FIPS—Freiburg index of post-TIPS survival.

The majority of patients (69.2%) suffered from ALD. The second most common cause of liver cirrhosis was NASH, followed by autoimmune hepatitis. Most patients were classified as Child–Pugh B and suffered from ascites and relevant esophageal or gastral varicosis. Encephalopathy was less prevalent, with 9.6%.

### Therapeutic results of the TIPS procedure

The TIPS procedures were performed between April 2010 and April 2021.

In two-thirds of cases, TIPS was performed due to therapy-refractory ascites. The second most common cause was secondary prevention of variceal bleeding. Overall, the majority of procedures (n = 49) were elective.

After TIPS implantation, the pressure gradients dropped from 21.9 ± 3.8 to 9.8 ± 3.1 mmHg, meeting the recommended target pressure gradient of < 12 mmHg^24^. In nine patients (17.3%), a revision had to be performed to dilate the stent or stent graft further. In follow-up imaging, ascites was resolved in 57.7% of cases.

Due to a lack of imaging, 35 patients also treated in our department could not be included in this retrospective analysis. Of these, only four patients were lost to follow-up within a year but at least 6 months after the TIPS procedure. Baseline data on these patients is provided in Supplementary Material Table 1.

### Increase of muscle area and decrease of sarcopenia after TIPS

In the majority of patients (74.0%), we analyzed CT images obtained at baseline and after a follow-up of at least six months and a median follow-up of 16.5 months. In a quarter of patients (26.0%), we analyzed MR images.

There was a positive correlation between the psoas muscle area delineated by hand and the semi-automated segmentation of the psoas muscle area (r = 0.773, *p* < 0.001).

There was no correlation between SMI and PMI before TIPS (r = 0.26; *p* = 0.11); however, we found a positive correlation after TIPS (r = 0.564; *p* < 0.001).

**Figure 1:**
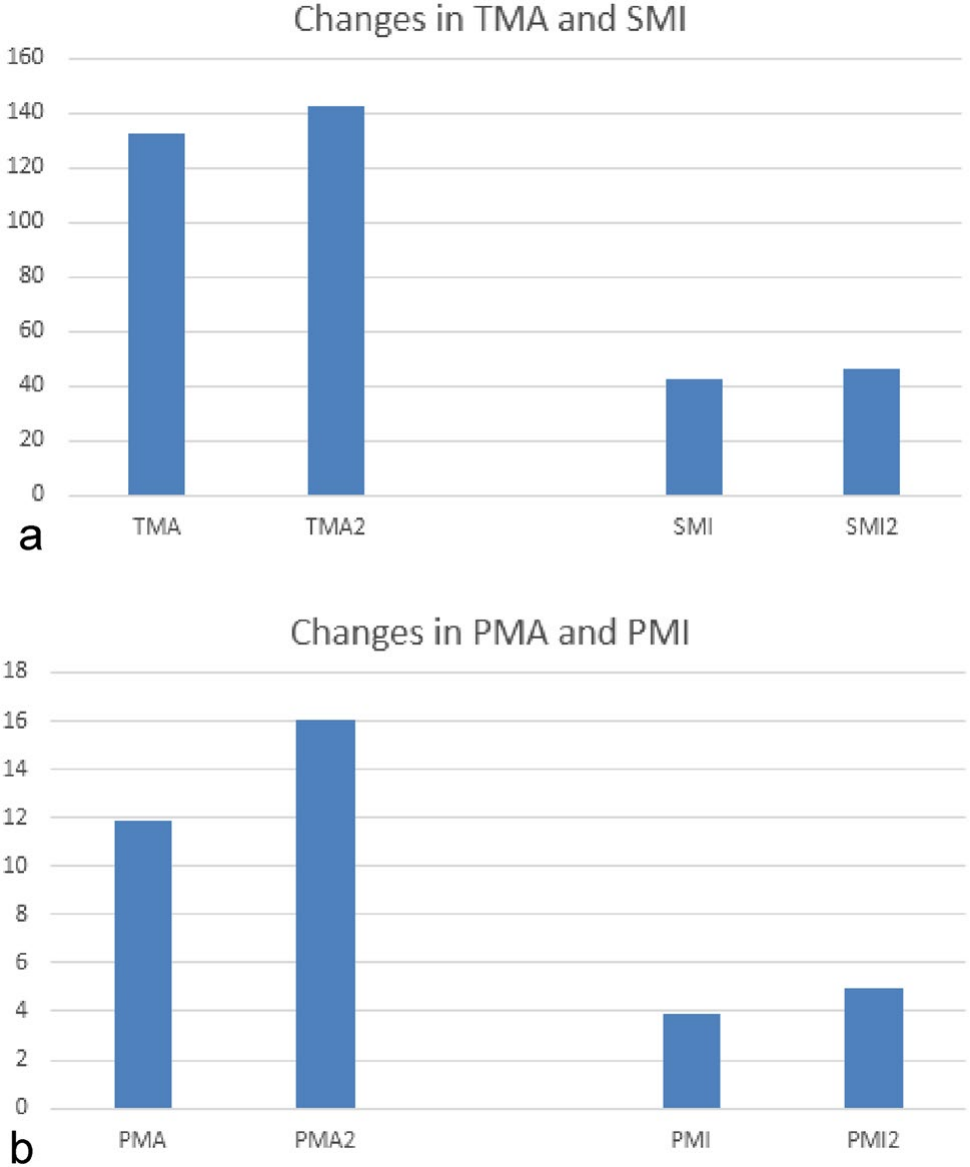
Changes in muscle area and muscle indices. (**a**) Changes in the total muscle area (TMA) and skeletal muscle index (SMI) before and after TIPS procedure (TMA2 and SMI2). (**b**) Changes in the psoas muscle area (PMA) and psoas muscle index (PMI) before and after TIPS procedure (PMA2 and PMI2).

**Figure 2:**
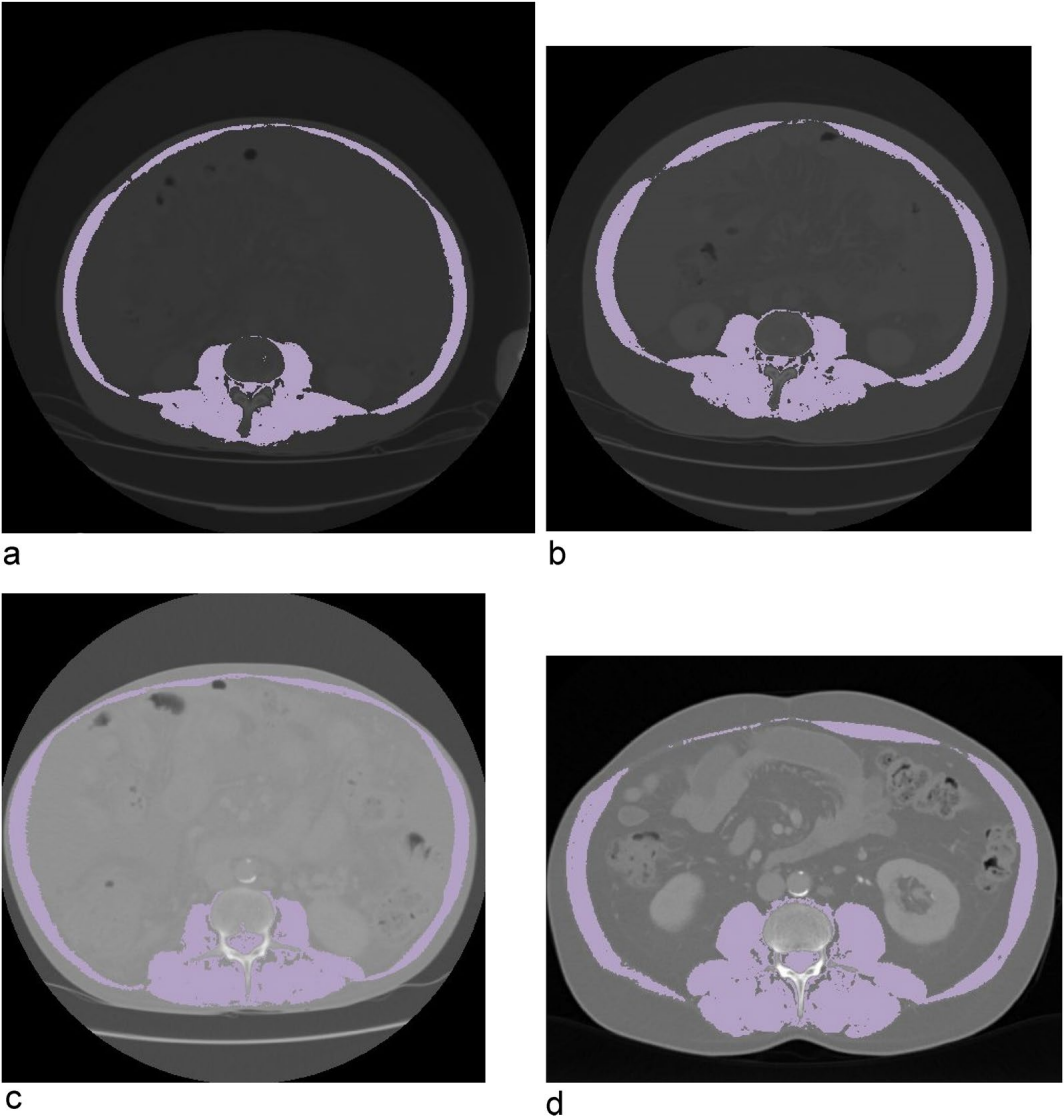
Changes in muscle indices by two examples. Upper row: at baseline (**a**) and 18 months follow-up (**b**) SMI: 48.0 versus 61.1 cm^2^/m^2^; Lower row: at baseline (**c**) and 36 months follow-up (**d**) SMI: 43.9 versus 57.9 cm^2^/m^2^.

After TIPS, a mean increase of 9.7 cm^2^ in TMA (*p* = 0.016) and of 4.1 cm^2^ in PMA (*p* < 0.001) was observed (Table 2 and Figs. 1a,b and 2). This resulted in significant increases of SMI (0.020) and PMI (*p* < 0.001).

**Table 2:** Patient characteristics before and after TIPS procedure.

n = 52	Preinterventional	Postinterventional	*P*
MELD	12.8 ± 4.6 (6–23)	13.4 ± 6.7 (7–23)	0.616
Bilirubin (qmol/l)	40.6 ± 68.5 (3.2–395.8)	65.5 ± 124.2 (3.7–643.0)	0.234
Quick (%)	72.5 ± 21.4 (26–120)	73.9 ± 24.6 (20–120)	0.617
Creatinine (qmol/l)	104.9 ± 52.1 (24–356)	123.3 ± 108.2 (27–681)	0.179
Albumin (g/l)	30.0 ± 7.0 (1.9–44.0)	33.1 ± 8.7 (12.3–50.1)	0.091
PMA (cm^2^)	11.9 ± 3.2 (6.2–22.1)	16.0 ± 8.2 (5.3–58.7)	< 0.001
TMA (cm^2^)	132.9 ± 21.3 (87.8–203.2)	142.6 ± 40.5 (72.7–227.0)	0.016
PMI (cm^2^/m^2^)	3.88 ± 0.9 (2.4–6.5)	4.95 ± 1.6 (1.5–8.7)	< 0.001
SMI (cm^2^/m^2^)	42.8 ± 6.4 (29.6–64.1)	46.4 ± 11.3 (25.8–71.6)	0.020

Results are presented as mean ± SD (range).

MELD—Model for end-stage liver disease; PMA—the area of right and left M. psoas; TMA—total muscle area; PMI—psoas muscle index; SMI—skeletal muscle index.

For the classification of possible sarcopenia, we applied previously used cut-off values for the SMI (39.5 cm^2^/m^2^ for women, ≤ 52 cm^2^/m^2^ for men)^25^ and the PMI (≤ 6.36 cm^2^/m^2^ in men ≤ 3.92 cm^2^/m^2^ in women)^26^. According to these cut-off values, most patients were classified sarcopenic (84.6%, 92.3%) at baseline. After the TIPS procedure, the proportion of patients with sarcopenia declined to 69.8% (SMI, *p* = 0.109) and 68.1% (PMI, *p* = 0.004). In a subgroup analysis of male patients, we found a significant decrease in sarcopenia according to the SMI (*p* = 0.039) and PMI (*p* = 0.008). There were no significant changes in muscle areas/indices in patients not classified as sarcopenic before the TIPS procedure (e.g., PMA, *p* = 0.50).

We did not evaluate weight and BMI since the majority of patients had relevant ascites, and weight information after drainage of ascites was often not available.

Analyzing patients with ascites after TIPS based on follow-up imaging, we found a significantly lower psoas muscle area (15.5 vs. 12.7 cm^2^, *p* = 0.037) and lower PMI (5.1 vs. 4.2 cm^2^/m^2^, *p* = 0.045) compared to patients without ascites.

Patients with ascites still showed some increase of muscle area after TIPS (TMA 135.4 vs. 138.7 cm^2^, *p* = 0.281; PMA 11.9 vs. 13.0 cm^2^, *p* = 0.184), but these results were not statistically significant.

### MELD and FIPS

On average, the MELD score before TIPS was 12.8. There was a slight increase to 13.4 after TIPS (Table 2). The average FIPS was − 0.263 prior to the TIPS procedure. According to the FIPS, overall survival one month after TIPS was estimated at 95.0%, 85.6% after three months, and 80.1% after six months. Before TIPS, there were three high-risk patients according to the cut-off value of ≥ 0.92 and nine high-risk patients with a FIPS higher than ≥ 0.64. In our study cohort, ascertained overall survival six months after TIPS was 100.0%.

As MELD and FIPS are based on similar parameters, there was a positive correlation between both scores (r = 0.513; *p* < 0.001), and the MELD in high-risk patients was significantly elevated (*p* = 0.001).

The MELD after TIPS was also inversely correlated to PMA (r = − 0.296, *p* = 0.039) and PMI after TIPS (r = − 0.536, *p* < 0.001).

However, we found no correlation between FIPS and muscle area/indices or significant differences between patients with a high and average risk of reduced post-TIPS survival (Suppl. Mat. Table S1).

Analyzing parameters for calculations of MELD and FIPS separately, we found no significant differences between patients classified as sarcopenic and non-sarcopenic. There was a slight, but not significant increase in albumin levels comparing all patients before and after TIPS (*p* = 0.091) (Table 2).

Interestingly, we found significantly higher serum albumin levels in patients with increased SMI compared to patients with no increase after TIPS (36.0 vs. 28.7; *p* = 0.022).

### Analysis according to the origin of liver cirrhosis

We analyzed differences in muscle area and indices between subgroups of patients according to the origin of the liver cirrhosis (Suppl. Mat. Table S2 and Fig. S1). For computational reasons, we formed four groups: ALD, NASH, autoimmune hepatitis, and Other. The group `Other` comprises different entities with one patient each: primary sclerosing cholangitis (PSC), primary biliary cholangitis (PBC), Budd-Chiari syndrome, and unknown.

**Figure 3:**
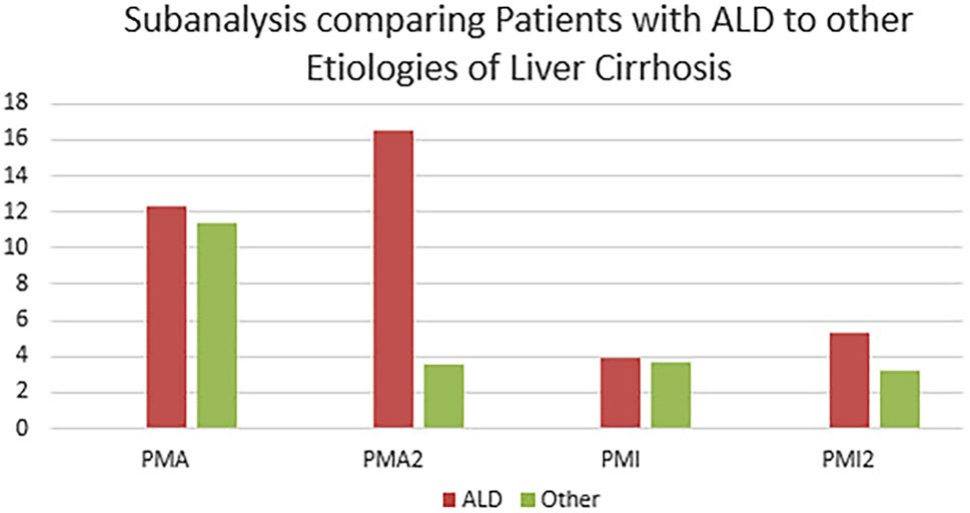
Subanalysis comparing patients with ALD to other etiologies of liver cirrhosis regarding changes in the psoas muscle area and psoas muscle index before (PMA, PMI) and after TIPS (PMA2, PMI2).

Comparing patients with alcoholic liver disease to all other patients, we found significant differences in the PMA (16.5 vs. 9.7; *p* = 0.006) and PMI (5.3 vs. 3.3; *p* = 0.016) after TIPS with no significant differences before the TIPS procedure (Table 3 and Fig. 3). Additionally, there was a significantly greater change in PMA in patients with alcoholic liver disease than in all other patients (5.7 vs. 1.1; *p* = 0.041).

**Table 3:** Subanalysis comparing patients with ALD to other etiologies of liver cirrhosis.

	Etiology	ALD	All other	*p*
TMA (cm^2^)		134.5 ± 20.3 (109.4–203.2)	125.3 ± 21.6 (87.8–158.3)	0.541
TMA2 (cm^2^)		160.8 ± 39.8 (88.6–227.0)	124.9 ± 24.3 (88.9–155.4)	0.355
PMA (cm^2^		12.3 ± 3.1 (7.2–19.1)	11.8 ± 2.7 (8.6–17.3)	0.367
PMA2 (cm^2^)		16.5 ± 5.2 (6.4–29.3)	9.7 ± 2.5 (5.3–13.0)	0.006
PMI (cm^2^/m^2^)		3.9 ± 1.0 (2.5–5.9)	3.8 ± 0.9 (2.6–5.5)	0.764
PMI2 (cm^2^/m^2^)		5.3 ± 1.5 (2.1–8.7)	3.3 ± 0.9 (1.5–4.1)	0.016
SMI (cm^2^/m^2^)		42.9 ± 5.8 (33.3–64.1)	42.0 ± 7.5 (29.4–53.5)	0.920
SMI2 (cm^2^/m^2^)		51.1 ± 11.7 (28.6–71.6)	41.5 ± 5.8 (34.6–50.6)	0.108

TMA—Total muscle area preinterventional; PMA—the area of right and left M. psoas preinterventional; PMI—psoas muscle index preinterventional; SMI—skeletal muscle index preinterventional, TMA2—total muscle area postinterventional; PMA2—the area of right and left M. psoas postinterventional; PMI2—psoas muscle index postinterventional; SMI2—skeletal muscle index postinterventional.

## Discussion

According to current studies, the prevalence of sarcopenia in patients with liver cirrhosis is estimated to be 25–45%^8^, depending on the method applied. At baseline, most patients in our study cohort were classified sarcopenic, with 85–92% depending on the used index system. As sarcopenia and malnutrition are associated with higher morbidity and mortality^6,8^, there is a need for therapeutic approaches to increase muscle quantity and quality.

Several studies have reported a positive correlation between TIPS implantation and an increase in muscle quantity^13–19^, along with indications for enhanced overall survival^10^. We evaluated muscle area and muscle indices in patients with liver cirrhosis by analyzing PMA and TMA in CT and MR images before and at least six months after the TIPS procedure.

Patients in our study cohort showed significant increases in TMA, PMA, SMI, and PMI. However, comparing patients with and without ascites after TIPS in follow-up imaging, patients with remaining ascites demonstrated significantly lower PMA and PMI, but increases in these parameters were still present, albeit not significant.

After TIPS, significantly fewer patients, including three patients with remaining ascites, were classified sarcopenic according to the PMI, and male patients were classified significantly less sarcopenic according to the SMI after the TIPS procedure.

It is suggested that the positive effect of a TIPS procedure on muscle quantity might be due to the deceleration of a catabolic metabolism, which is caused by proinflammatory cytokines, leading to loss of muscle mass^27^, the reversal of portal hypertension and aftereffects such as ascites^13^. This is also indicated by our results, as patients with resolved ascites after TIPS showed the greatest improvement regarding muscle quantity and reduction of sarcopenia.

As the function of hepatocytes in liver cirrhosis is impaired, so is the synthesis of albumin^28^. Albumin is solely produced in the liver parenchyma and provides an anti-inflammatory effect by transporting toxic metabolites, such as bilirubin and acids. It also serves as an antioxidant. Long-term administration of albumin reduces complications and improves survival^29^. We analyzed changes in albumin, bilirubin, and creatinine and found a slight, albeit not significant, increase in albumin levels after the TIPS procedure. However, we found a significant elevation in albumin levels in patients with increased SMI after TIPS compared to patients without an increase after the TIPS procedure. Our findings might indicate a metabolic shift in patients with increased muscle mass after TIPS implantation. Further studies are needed to evaluate the possible effects of albumin on or as a biomarker for muscle quantity.

We found no positive effect of TIPS on muscle area and indices in patients not classified as sarcopenic before the TIPS procedure. This is in line with findings of Liu et al.^10^.

ALD was the most common etiology of liver cirrhosis in our study cohort. Patients with ALD seem to exhibit the lowest muscle area compared to other etiologies of liver cirrhosis and also show a higher rate of muscle depletion^11^. We evaluated muscle area and indices between subgroups of patients with different etiologies of liver cirrhosis. Before the TIPS procedure, we found no significant differences between patients with ALD and all other patients. However, after the TIPS procedure, we found a significantly higher PMA and PMI in patients with ALD than in all other patients. To our knowledge, this has not been reported before. A possible explanation might be additional lifestyle changes in patients with ALD with the abstain from alcohol.

We retrospectively applied the FIPS to analyze the likelihood of post-TIPS survival. We identified three high-risk patients with FIPS ≥ 0.92 and nine high-risk patients with FIPS ≥ 0.64. As expected, the MELD in these patients was significantly elevated, as there was a strong correlation between MELD and FIPS. Ascertained survival six months after TIPS was 100.0%.

We found no significant differences between high-risk and average-risk patients regarding muscle area and indices before and after TIPS. A possible explanation could be the relatively small number of high-risk patients. However, there was a strong negative correlation between MELD after TIPS and PMI after TIPS, implicating a higher likelihood of survival in patients with higher psoas muscle index after TIPS.

### Limitations

This is a retrospective study in an uncontrolled study design, analyzing patients over a period of 11 years. Changes in the therapeutic management of liver cirrhosis are likely, which makes for a heterogenic study group. As the majority of patients undergoing TIPS procedures at our Department did not receive CT or MR imaging, the sample size is limited.

Using CT as well as MR images could hamper the data quality or comparability, as it is not undisputed if measurements in CT and MR images are interchangeable and most studies on muscle quantity are solely based on CT images. However, this seems not likely, as the majority of analysed images were CTs and two different imaging analysis methods with a strong correlation of results were used.

The use of cut-off values for sarcopenia in existing studies is very heterogeneous. We decided to apply cut-off values, which have been used on a predominantly European study group^25^, reflecting our cohort's patient demography.

Most importantly, as with most studies on sarcopenia in liver cirrhosis, we only evaluated muscle quantity, not quality. Further studies are needed to evaluate not only muscle quantity but also muscle function.

## Conclusions

In summary, our study confirms previous studies, demonstrating a positive impact of a TIPS procedure on muscle area and indices in patients with liver cirrhosis who were classified as sarcopenic. Patients with resolved ascites and alcoholic liver disease seem to profit the most. In addition, we found higher albumin levels in patients with increased SMI after TIPS compared to patients without an increase. A possible association between albumin and muscle quantity needs further evaluation.

### Supplementary Information

Supplementary Information.

## Data Availability

The datasets generated during and/or analysed during the current study are available from the corresponding author on reasonable request.

## Abbreviations

ALD: Alcoholic liver disease
CT: Computed-tomography
EWGSOP: European Working Group on Sarcopenia in Older People
FIPS: Freiburg index of post-TIPS survival
HCC: Hepatocellular carcinoma
HCV: Hepatitis C virus
HBV: Hepatitis B virus
HU: Hounsfield units
LSMM: Low skeletal muscle mass
NAFLD: Non-alcoholic liver disease
NASH: Nonalcoholic steatohepatitis
MELD: Model for end-stage liver disease
MRI: Magnetic resonance imaging
PBC: Primary biliary cirrhosis
PMA: Area of right and left M. psoas
PMI: Psoas muscle index
PSC: Primary sclerosing cirrhosis
SMI: Skeletal muscle index
SMM: Skeletal muscle mass
SFA: Skeletal fat area
TFA: Total fat area
TIPS: Transjugular intrahepatic portosystemic shunt
TMA: Total muscle area
TMFA: Total muscle fat area
VFA: Visceral fat area
