# Supplementary material for: Increase of radiologically determined muscle area in patients with liver cirrhosis after transjugular intrahepatic portosystemic shunt
Source: Sci Rep. 2023 Oct 10;13:17092. doi: 10.1038/s41598-023-43938-6 (PMC10564886; doi:10.1038/s41598-023-43938-6)
Supplement: Supplementary file 1 — Supplementary Information. [file 41598_2023_43938_MOESM1_ESM.docx]

**Increase of radiologically determined muscle area in patients with liver cirrhosis after transjugular intrahepatic portosystemic shunt**

Christine March^1^, Maximilian Thormann^1^, Sarah Geipel^1^, Jan-Peter Sowa^2^, Felix Barajas Ordonez^1^, Maciej Pech^1^, Jazan Omari^1*^, Peter Lemmer^3*^

**Table of content**

- Table 1: Characteristics of 35 patients who had undergone TIPS procedure – omitted from the study.
- Figure 1: Subanalysis according to the origin of liver cirrhosis
- Table 1: Comparison of normal and high risk patients according to FIPS
- Table 2: Subanalysis according to the etiology of liver cirrhosis

**Table 1: Characteristics of 35 patients who had undergone TIPS procedure – omitted from the study.**

|  | n = 35 |
| --- | --- |
| Sex |  |
| Male, % | 23 (65.7) |
| Female, % | 12 (34.3) |
| Age (years), mean ± SD (range) | 56.6 ±11.0 (32-81) |
| Ascites, % | 34 (97.1) |
| Indication TIPS |  |
| Therapy-refractory ascites, % | 32 (91.4) |
| Variceal hemorrhage, % | 2 (5.7) |
| Other, % | 1 (2.9) |
| Urgency |  |
| Emergency, % | 2 (5.7) |
| Elective, % | 33 (94.3) |
|  |  |

NASH - nonalcoholic steatohepatitis; TIPS - transjugular intrahepatic portosystemic shunt

**Fig. S1**: Subanalysis according to the origin of liver cirrhosis

TMA - total muscle area preinterventional; TMA2 - total muscle area postinterventional; PMA – the area of right and left M. psoas preinterventional; PMA2 – the area of right and left M. psoas postinterventional; PMI - psoas muscle index preinterventional; PMI2- psoas muscle index postinterventional; SMI - skeletal muscle index preinterventional, SMI2 - skeletal muscle index postinterventional; ALD – alcoholic liver disease; NASH – nonalcoholic steatohepatitis

**Table S1: Comparison of normal and high risk patients according to FIPS.**

| **FIPS** | **Normal risk** | **High risk** | **P** |
| --- | --- | --- | --- |
| PMA (cm²) | 12.3 (±2.8) | 13.1 (±5.4) | 0.502 |
| TMA (cm²) | 133.9 (±15.9) | 141.1 (±58.2) | 0.638 |
| PMI (cm²/m²) | 4.0 (±0.9) | 4.1 (±1.6) | 0.158 |
| SMI (cm²/m²) | 43.7 (±4.7) | 44.8 (±17.7) | 0.638 |
| MELD | 11.8 (±3.6) | 18.7 (±3.1) | 0.001 |

FIPS - Freiburg index of post-TIPS survival, BMI – body mass index; PMA – the area of right and left M. psoas; TMA - total muscle area; PMI - psoas muscle index; SMI - skeletal muscle index; MELD - Model for End-Stage Liver Disease

**Table S2: Subanalysis according to the etiology of liver cirrhosis.**

|  | Etiology | | ALD | NASH | Autoimmune Hepatitis | Other |  |
| --- | --- | --- | --- | --- | --- | --- | --- |
| TMA (cm²) | |  | 134.5±20.3 | 146.9±16.0 | 133.9±0.9 | 110.3±18.5 |  |
| TMA2 (cm²) | |  | 160.8±39.8 | 130.4±27.5 | 138.1±24.5 | 115.6±25.9 |  |
| PMA (cm²) | |  | 12.3±3,1 | 11.4±0.2 | 10.2±1.3 | 12.7±3.6 |  |
| PMA2 (cm²) | |  | 16.5±5.2 | 10.7±1.2 | 7.1±2.6 | 10.6±2.5 |  |
| PMI (cm²/m²) | |  | 3.9±1.0 | 3.9±0.1 | 3.2±0.8 | 4.1±1.1 |  |
| PMI2 (cm²/m²) | |  | 5.3±1.5 | 3.7±3.2 | 2.3±1.1 | 3.6±0.6 |  |
| SMI (cm²/m²) | |  | 42.9±5.8 | 50.4±4.2 | 41.9±5.9 | 37.8±6.6 |  |
| SMI2 (cm²/m²) | |  | 51.1±11.7 | 42.7±8.4 | 44.7±1.9 | 39.2±6.3 |  |

TMA - total muscle area preinterventional; PMA – the area of right and left M. psoas preinterventional; PMI - psoas muscle index preinterventional; SMI - skeletal muscle index preinterventional, TMA2 - total muscle area postinterventional; PMA2 – the area of right and left M. psoas postinterventional; PMI2- psoas muscle index postinterventional; SMI2 - skeletal muscle index postinterventional
